# Bariatric surgery in liver cirrhosis

**DOI:** 10.3389/fsurg.2022.986297

**Published:** 2022-12-15

**Authors:** A. S. Mehdorn, Y. Moulla, M. Mehdorn, A. Dietrich, W. Schönfels, T. Becker, F. Braun, J. H. Beckmann, M. Linecker

**Affiliations:** ^1^ Department of General, Visceral, Thoracic, Transplantation and Pediatric Surgery, University Hospital Schleswig-Holstein Campus Kiel, Kiel, Germany; ^2^ Department of Visceral, Transplant, Thoracic, and Vascular Surgery, University Hospital of Leipzig, Leipzig, Germany

**Keywords:** bariatric surgery, sleeve gastrectomy, obesity, obese patients, liver cirrhosis, NAFLD, Roux-en-Y-gastric bypass, liver failure

## Abstract

**Introduction:**

Obesity is frequently associated with its hepatic manifestation, the nonalcoholic fatty liver disease (NAFLD). The most effective treatment for morbid obesity is bariatric surgery (BS) also improving NAFLD and liver function. In patients where NAFLD has already progressed to liver cirrhosis, BS can be considered a high-risk procedure. Hence, consideration of the procedure and the most appropriate timing is crucial.

**Material and Methods:**

Obese patients suffering from NAFLD who underwent BS from two German University Medical Centers were retrospectively analyzed.

**Results:**

Twenty-seven patients underwent BS. Most common procedures were laparoscopic Roux-en-Y-gastric (RYGB) and laparoscopic sleeve gastrectomy (SG). All patients suffered from liver cirrhosis Child A. A preoperative transjugular portosystemic shunt (TIPS) was established in three patients and failed in another patient. Postoperative complications consisted of wound healing disorders (*n *= 2), anastomotic bleeding (*n *= 1), and leak from the staple line (*n *= 1). This patient suffered from intraoperatively detected macroscopic liver cirrhosis. Excess weight loss was 73% and 85% after 1 and 2 years, respectively. Two patients suffered from postoperative aggravation of their liver function, resulting in a higher Child–Pugh score, while three could be removed from the waiting list for a liver transplantation.

**Conclusion:**

BS leads to weight loss, both after SG and RYGB, and potential improvement of liver function in liver cirrhosis. These patients need to be considered with care when evaluated for BS. Preoperative TIPS implantation may reduce the perioperative risk in selected patients.

## Introduction

Consumption of a high caloric diet combined with a sedentary lifestyle has led to a significant rise of obesity worldwide (1, 2). In 2019, 2 billion people were assumed to be obese with increasing numbers (3). Obesity is embedded in a syndromic complex along with arterial hypertension, diabetes mellitus/insulin resistance, and dyslipidemia, called the metabolic syndrome (MES) (1, 2, 4, 5). The hepatic manifestation of the MES is nonalcoholic fatty liver disease (NAFLD), potentially causing nonalcoholic steatosis hepatis (NASH) and liver cirrhosis, followed by liver failure and eventually development of hepatocellular carcinoma (HCC) and other cancers (2, 6, 7). In females, NASH has meanwhile become the most common reason for liver failure and numbers in males are very close (1, 7, 8).

Liver transplantation (LT) is the only curative treatment for patients suffering from liver failure, particularly when presenting with a high model of end stage liver disease (MELD) score. Morbidly obese patients waiting for an LT do have a markedly increased risk of perioperative complications, e.g., portal vein thrombosis, bleeding, and increased risk for infectious complications (9–11). As a consequence, these patients are frequently not listed for LT or even delisted (i.e., removed from waiting list) for being too sick (10, 12–15). In addition, performing LT in obese patients is technically challenging and associated with longer operating times, more reoperations, and an increased complication rate (9, 16). This is the reason why LT was previously considered a contraindication in morbid obesity (17).

Normalization of body weight and reduction of obesity-associated comorbidities may even avoid LT as NASH and NASH-associated consequences, i.e., liver fibrosis and cirrhosis, may be positively influenced by weight reduction (4, 18). Unfortunately, conservative therapy for obesity, MES, and NAFLD, i.e., life style modifications and drug treatments, is often of little success (1, 4, 18, 19). Bariatric surgery (BS), on the other hand, is a highly effective and meanwhile well-established form of treatment for obesity and obesity-associated comorbidities, particularly NAFLD (6, 18, 20). Nowadays, BS is mainly performed minimally invasive, markedly reducing postoperative complications and sequelae compared to open surgery (16, 21, 22). Currently, the two main bariatric procedures are sleeve-gastrectomy (SGs) and Roux-en-Y-gastric (RYGBs).

Obese patients suffering from liver failure need to be considered high-risk patients for postoperative morbidity and mortality in any type of surgery (16, 21). Hence, in a multistep approach, BS may be performed primarily in order to improve NASH and liver function after failure of conservative approaches, potentially avoiding a LT.

The aim of our retrospective research was to assess the outcome of morbidly obese patients suffering from NAFLD-induced liver cirrhosis undergoing BS at two German reference centers for bariatric surgery.

## Material and methods

### Centers

Participating centers were University Medical Center Schleswig-Holstein, Campus Kiel, and the University Medical Center Hospital Leipzig, Germany. Both centers are board-qualified reference centers for bariatric surgery. Local ethics committees had given written approval (D513/16, 157-12-12122011). All patients included had given written informed consent for participation. Data were retrieved retrospectively from in-house patient files. Only deidentified data were used and data were handled according to the Declaration of Helsinki.

### Study population and inclusion/exclusion criteria

Obese patients [Class II, body mass index (BMI) > 35 kg/m^2^] scheduled for BS from 2013 to 2021 suffering from concomitant NAFLD-induced cirrhosis were included in this study. Some patients suffered from known NAFLD cirrhosis and were, therefore, scheduled for BS in order to improve liver function and prevent failure, while in other patients cirrhosis was an incidental finding intraoperatively or during protocol biopsy. Some patients were already on the waiting list for a LT. Excluded were obese patients suffering from other forms of liver.

### Bariatric surgery

BS was indicated according to the German guidelines for bariatric surgery (20). All patients without primary indication for BS had undergone at least a 6-month multimodal conservative treatment, consisting of supervised physical activity and nutrition counseling, followed by psychiatric evaluation and gastroscopy in preparation for BS. BS was performed laparoscopically in both centers and consisted of SG and RYGB as previously described (21, 22).

Patients were positioned in the French position and received calf-compression pumps. For both procedures, a capnoperitoneum was established at 15 mmHg. In all patients, a protocol liver biopsy was taken. For SG, a 40F bougie was introduced transorally after dissection of the gastroepiploic vessels at the great curvature. The greater curvature was then transected using a 45 mm ECHELON™ staple (Ethicon, Raritan, NJ, United States) (21). The staple line was overstitched in one center by a running suture using 4.0 Stratafix™ (Ethicon, Raritan, NJ, United States). For the RYGB, a small gastric pouch was created using the 45 mm ECHELON staple (Ethicon, Raritan, NJ, United States). After placing a 40F bougie, an alimentary limb (150–170 cm) and a biliary limb (50–100 cm) were created and stapled using the 45 mm ECHELON staple (Ethicon, Raritan, NJ, United States). The defects were sutured using 4.0 Stratafix™ (Ethicon, Raritan, NJ, United States). Blue test of the gastrojejunostomy was performed. Drains were placed for both procedures. Patients were extubated directly after surgery and mobilization was mandatory on the day of surgery. Patients received intravenous and oral fluids starting the day of surgery. Oral fluid and food intake was increased within days after the surgery. All patients received postoperative nutritionist counseling. Drains were removed before discharge. Prophylactic antithrombotic prophylaxis was applied according to the body weight. Regular surgical and nutritionist follow-ups were mandatory, including checks for electrolytes, vitamin, and trace elements. Perioperative treatment was conducted as previously described (21).

### Primary and secondary outcome measures

Primary outcome measures were effectiveness of BS (excessive weight loss, EWL) and improvement of liver function (MELD, Child–Pugh). Secondary outcome measures were complications.

### Statistics

Normally distributed continuous variables were presented as mean (±SD), and in median (range). Categorical variables were presented in absolute numbers and in percent (%) of the total number. In univariate analysis, groups were compared using the two-sided Student’s *t*-test for continuous variables. Chi-square test was used for categorical variables. Statistics were performed using GraphPad Prism version 9.3.1 (350) (GraphPad Software, San Diego, CA, United States) and Statistical Package for Social Sciences (SPSS) 28.0.0.0 (190) (IBM, Armonk, NY, United States) for Mac.

## Results

**Figure 1:**
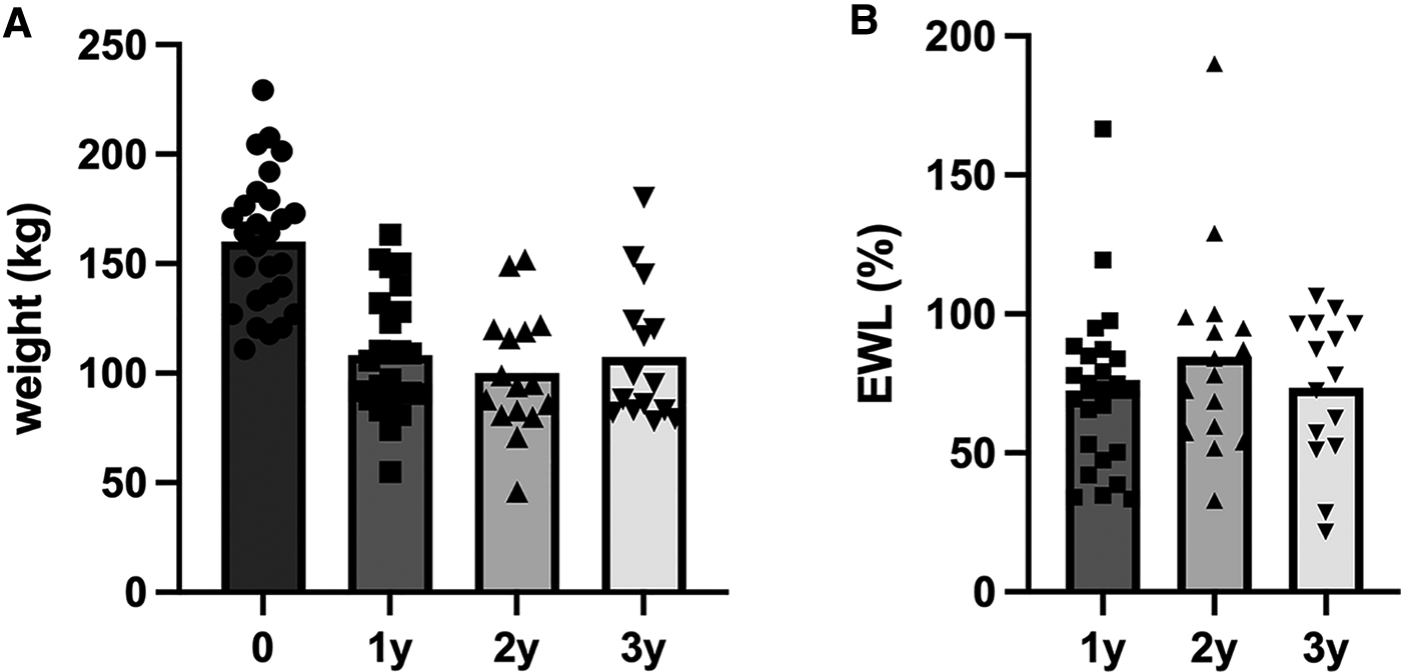
Development of weight of patients undergoing bariatric surgery suffering from liver cirrhosis (*n *= 27). Weight (kg) at time of surgery (0), 1 year (1y), 2 years (2y), and 3 years (3y) after surgery (**A**). Excess weight loss [EWL (%)] after 1, 2, and 3 years (**B**).

Twenty-seven patients met the inclusion criteria and were included in the final analysis (Table 1). Patients were on average 51.8 ± 9.6 years old with the majority being female. Preoperative BMI was 51.7 ± 10.2 kg/m^2^ and MELD at the time of BS was 15 ± 5. All patients suffered from NAFLD cirrhosis, clinically classified as Child–Pugh A-cirrhosis. Grade of cirrhosis was missing in three cases. In 21 patients, liver cirrhosis had been an incidental finding during protocol biopsy during BS. Three additional patients were listed for LT, and BS was performed during the preparation for LT. Patients suffered on average of 4 ± 1 comorbidities, mainly arterial hypertension (85%) and diabetes mellitus (78%). A preoperative transjugular portosystemic shunt (TIPS) was successfully placed in three patients and failed in another one. The most common BS procedure was RYGB (*n *= 14, 52%), followed by SG (*n *= 10, 37%). No intraoperative complications were noticed, but two patients experienced Clavien–Dindo III and V complications: One patient suffered from an anastomotic bleeding requiring revisional surgery. The other patient developed an intra-abdominal abscess probably due to a leak from the staple line after SG. This patient had a complicated postoperative course and died due to respiratory distress 30 days after surgery. Two patients suffered from postoperative wound healing. EWL after 1, 2, and 3 years was 73% (range: 33%–167%), 85% (range: 33%–190%), and 73% (29%–107%) (Figure 1). In two patients, one after SG and one after RYGB, we noticed impaired liver function 1 year after BS. Most patients, however, showed a steady state regarding the Child–Pugh score value. Three patients could be delisted from the waiting list for an LT due to improved liver function.

**Table 1:** Patients undergoing BS suffering from liver cirrhosis (*n* = 27).

BS in patients suffering from liver cirrhosis	
Age at BS (years, mean ± SD)	52 ± 10
Sex (*n*, % males)	11, 40.7
BMI prior to BS (kg/m^2^, mean ± SD)	52 ± 10
BW prior to BS (kg, mean ± SD)	160 ± 31
MELD at BS (mean ± SD)	5 ± 5
Child Pugh at BS (A/B/C) (*n*, %)	27/0/0, 100/0/0
Preoperative Child–Pugh score (mean, range)	5 (5–6)
Number of comorbidities (*n*)	4 ± 1
Arterial hypertension (*n*, % yes)	23, 85
Diabetes mellitus (*n*, % yes)	21, 78
Arthrosis (*n*, % yes)	14, 52
GERD (*n*, % yes)	13, 48
Sleep apnea (*n*, % yes)	11, 41
Asthma/COPD (*n*, % yes)	8, 30
Hypothyreosis (*n*, % yes)	2, 7
Esophageal varices (*n*, % yes)	2, 7
Arterial fibrillation (*n*, % yes)	1, 4
Renal insufficiency (*n*, % yes)	1, 4
Preoperative TIPS (*n*, % yes)	3, 11^a^
Bariatric procedure (*n*, % yes)	
RYGB	14, 52
SG	10, 37
GB to RYGB	1, 4
RYGB to EGB	1, 4
Omega-Loop	1, 4
Length of BS (min ± SD)	138.4 ± 39.2
Intraoperative complications (*n*, % yes)	0, 0
Postoperative complications (*n*, % yes)	4, 15
Clavien–Dindo I	0.0
Clavien–Dindo II	2, 7
Clavien–Dindo III	1, 4
Clavien–Dindo IV	0.0
Clavien–Dindo V	1, 4
LOS after BS (days ± SD)	4.5 ± 0.8
Child–Pugh score at 1 year (A/B/C) (*n*, %)	24/0/2
1-year Child–Pugh score (mean, range)	5 (5–13)

BS, bariatric surgery; BMI, body mass index; BW, body weight; COPD, chronic obstructive pulmonary disease; EGB, extended laparoscopic gastric bypass; GERD, gastroesophageal reflux disease; GB, gastric bypass; SG, sleeve gastrectomy; LOS, length of stay; RYGB, Roux-en-Y-gastric bypass; MELD, model of end stage liver disease; SD, standard deviation; TIPS, transiugular portosystemic shunt.

^a^ Excluding one attempted/failed TIPS introduction.

**Figure 2:**
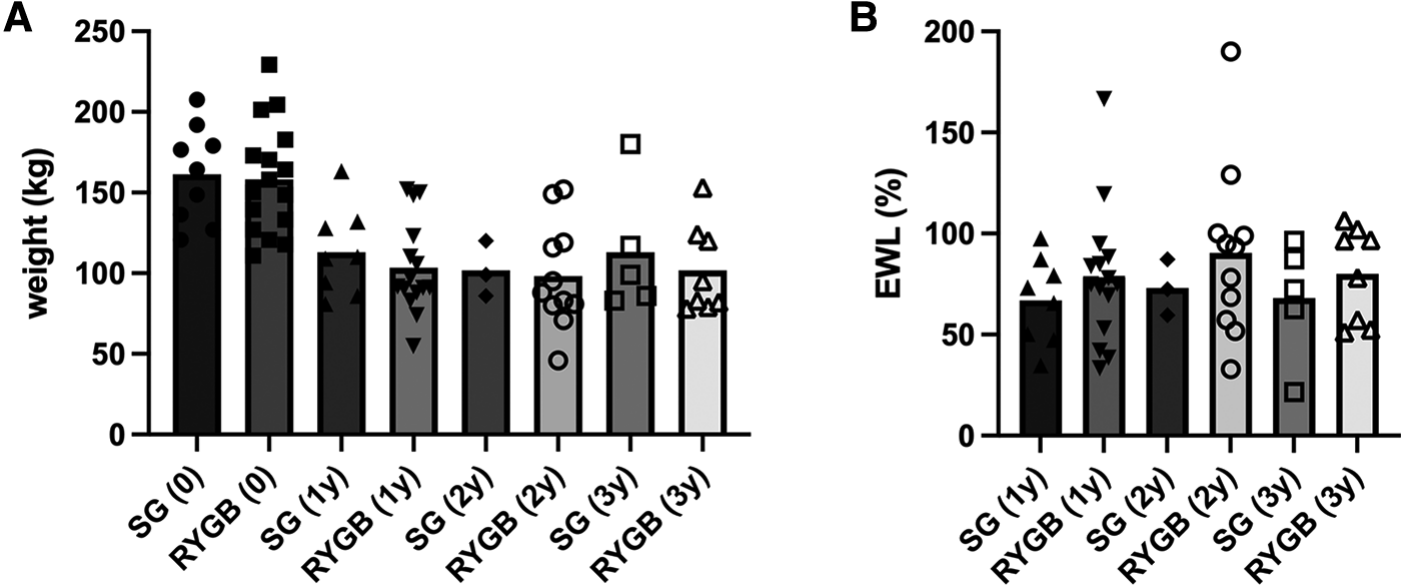
Development of weight and excess weight loss (EWL) of patients undergoing bariatric surgery suffering from liver cirrhosis, stratified by bariatric procedure. Weight (kg) at time of surgery (0), 1 year (1y), 2 years (2y), and 3 years (3y) after surgery (**A**). Excess weight loss [EWL (%)] after 1, 2, and 3 years (**B**). One patient was excluded due to another procedure type (Omega-Loop), impossible to fit into the comparison. SG, sleeve gastrectomy; RYGB, Roux-en-Y-gastric bypass.

Interprocedural (SG vs. RYGB) comparison of patients with liver cirrhosis undergoing BS revealed a significantly higher incidence of diabetes mellitus (60% vs. 94%, *p* = 0.034) and significantly longer procedures times (115 ± 40 min vs. 156 ± 33 min, *p* = 0.010) in the RYGB group (Table 2). Interestingly, patients receiving an RYGB were significantly older compared to patients receiving an SG (46 ± 8 years vs. 56 ± 9 years, *p* = 0.013). Of note, postoperative complications were noted in both groups. One anastomotic bleeding and one wound healing disorder were noted in the RYGB group. A leak from the staple line, clinically apparent *via* presentation of an abscess, followed by postoperative death 30 days after surgery and one wound healing disorder were noticed in the SG group. Both procedures achieved weight loss in the patients included (Figure 2). EWL [1- and 2-year-EWL% of 73% (range 33%–167%) and 85% (range 33%–190%)] was within known ranges, yet without showing superiority of one procedure over the other (Figure 2).

**Table 2:** Comparison of patients receiving BS stratified by bariatric procedure. One patient was excluded due to another procedure type (Omega-Loop), impossible to fit into the comparison.

	SG (*n *= 10)	RYGB (*n *= 16)	*p*-value
Age at BS (years, mean ± SD)	46 ± 8	56 ± 9	**0**.**013**^a^
Sex (*n*, % males)	3, 30	7, 44	0.483^b^
BMI prior to BS (kg/m^2^, mean ± SD)	53 ± 10	51 ± 11	0.553^a^
BW prior to BS (kg, mean ± SD)	162.0 ± 28.4	158.2 ± 34.1	0.770 ^a^
MELD at BS (mean ± SD)	14 ± 5	16 ± 4	0.361^a^
Child–Pugh score at BS (A/B/C) (*n*, %)	10/0/0, 100/0/0	16/0/0, 100/0/0	NA
Preoperative Child–Pugh score (mean, range)	5 (5)	5 (5–6)	0.082
Number of comorbidities (*n*)	3 ± 1	4 ± 1	0.083^a^
Arterial hypertension (*n*, % yes)	8, 80	15, 94	0.286^b^
Diabetes mellitus (*n*, % yes)	6, 60	15, 94	**0**.**034**^b^
Arthrosis (*n*, % yes)	5, 50	7, 56	0.756^b^
GERD (*n*, % yes)	5, 50	8, 50	1.000^b^
Sleep apnea (*n*, % yes)	3, 30	8, 50	0.315^b^
Asthma/COPD (*n*, % yes)	3, 30	5, 31	0.946^b^
Hypothyreosis (*n*, % yes)	1, 10	1, 6	0.727^b^
Esophageal varices (*n*, % yes)	1, 10	1, 6	0.727^b^
Arterial fibrillation (*n*, % yes)	0, 0	1, 6	0.420^b^
Renal insufficiency (*n*, % yes)	1, 10	0, 0	0.197^b^
Preoperative TIPS (*n*, % yes)	2^c^, 20	1, 6	0.433^b^
Length of BS (min ± SD)	114.8 ± 40.3	155.5 ± 32.5	**0**.**010**^a^
Intraoperative complications (*n*, % yes)	0, 0	0, 0	na
Postoperative complications (*n*, % yes)	2, 20	2, 12.5	0.392^b^
Clavien–Dindo I	0, 0	0, 0	
Clavien–Dindo II	1, 50	1, 50	
Clavien–Dindo III	0, 0	1, 50	
Clavien–Dindo IV	0, 0	0, 00	
Clavien–Dindo V	1, 50	0, 0	
LOS after BS (days ± SD)	4.8 ± 1.3	4.8 ± 0.5	0.241^a^
Child Pugh at 1 year (A/B/C) (*n*, %)	9/0/1	12/0/1	0.245
1-year Child–Pugh Score (mean, range)	6 (5–15)	5 (5–12)	0.642

BS, bariatric surgery; BMI, body mass index; BW, body weight; COPD, chronic obstructive pulmonary disease; GERD, gastroesophageal reflux disease; SG, sleeve gastrectomy; LOS, length of Stay; RYGB, Roux-en-Y-gastric bypass; MELD, model of end stage liver disease; SD, standard deviation; TIPS, transiugular portosystemic shunt.

^a^ Student’s *t*-test.

^b^ Chi-square test, significant p-values are marked in bold.

^c^ Excluding one attempted/failed TIPS introduction.

However, we have to report on a certain intercenter variability with one center performing more SG in patients with known liver cirrhosis after preparation with a TIPS, while the other center preferentially performed RYGB, reported on two redo-procedures and had significantly more (17/20) incidental intraoperative findings of liver cirrhosis.

## Discussion

In this German dual-center series, we report on our experience after BS in morbidly obese patients suffering from NAFLD cirrhosis. According to our data, BS seems to be a reasonable option with an acceptable perioperative risk in obese patients suffering from liver cirrhosis Child A. Both, RYGB and SG seem to be feasible and safe in these patients achieving satisfying weight losses with 1- and 2-year-EWL% of 73% (range 33%–167%) and 85% (range 33%–190%), respectively. Most patients included showed no aggravation of their liver cirrhosis during the follow-up period. Due to improved liver function, three patients could even be delisted from the waiting list.

Consistent with our cohort, most common bariatric procedures currently performed are SG and RYGB. SG was originally introduced for patients presenting with severe obesity, not suitable for RYGB (20, 23). Creation of the gastric sleeve is based on restriction, mainly preserving the original anatomy, thereby little influences blood supply of the liver itself and preserves endoscopic access to the biliary tract (1, 24). Additionally, due to the purely restrictive nature of the procedure, gastrointestinal absorption is only changed minimally while improving NAFLD (25). SG often has to deal with acid reflux, potentially causing Barrett disease and impairing patients’ quality of life (11). RYGB, on the other hand, combines a restrictive and a malabsorptive component coming along with major anatomical changes and metabolic reprogramming (26, 27). RYGB is hence considered to be more effective for patients suffering from diabetes mellitus, arterial hypertension, and other manifestations of the metabolic syndrome. It may have different absorption for immunosuppressive drugs in designated LT patients (28, 29).

Patients included in this study approached BS for two different indications: Some patients suffered from severe obesity presenting with the intraoperative, incidental finding of liver cirrhosis. These patients were often treated with RYGB due to other comorbidities. Other patients included suffered from known liver cirrhosis and therefore underwent BS, mainly SG. These two different approaches explain the heterogeneity in the procedures chosen. Taking EWL% as a main outcome parameter in BS into consideration, we could not identify a significant difference between the two types of surgery, considering both procedures equally effective in our cohort. RYGB is often seen to be more effective regarding weight loss and improvement of other comorbidities (6, 30).

Perioperative complications were comparable between both procedures, making both procedures technically safe in this selected cohort. Yet, exact estimation of preoperative surgical risk is crucial as BS in patients suffering from liver cirrhosis is considered to be a high-risk procedure due to impaired wound healing mechanisms and decreased immune function. Such a complication may further lead to a hepatic decompensation, a potentially life-threatening condition with the need of urgent liver transplantation (31). In this context, especially RYGB is estimated to come along with increased intra- and perioperative risk for complications (4). Child–Pugh classification, MELD score, and the presence of portal hypertension are helpful parameters to evaluate the preoperative risk of a patient (4, 18). In our cohort, Child–Pugh classification and MELD score were assessed in all patients; hepatic venous pressure gradient (HVPG) measurement and TIPS evaluation was only performed on demand when indirect signs of portal hypertension were present in patients with preoperatively known liver cirrhosis. These patients were scheduled for subsequent TIPS implantation indicating that suspicion of increased HVPG was highly specific. However, TIPS implantation is not always technically achievable. Due to the absence of a screening protocol for elevated HVPG, patients suffering from portal hypertension may be missed in the preoperative evaluation. However, currently, neither the German guideline for bariatric surgery nor the German guideline for NAFLD provides a clear recommendation for severe obese patients suffering from liver cirrhosis undergoing BS with regard to a preoperative TIPS implantation or evaluation (18, 20). In a recent consensus statement from the international federation of the surgery of obesity and metabolic disorders (IFSO) TIPS implantation prior to performing a SG in patients suffering from liver cirrhosis is recommended (oral communication).

Of greatest interest in obese patients suffering from NAFLD is not only EWL but also improvement of liver fibrosis and cirrhosis. In this regard, Lassailly et al. reported on 5-year-biopsy-proven remission of NASH and hepatic fibrosis in 180 patients after BS for severe obesity (32). Kim et al. as well report on good outcome in NAFLD patients after both procedures (33). de Brito E Silva report in their recently published review that both procedures are equally effective for treating NAFLD and NASH, but hepatic fibrosis is ameliorated to a greater extend after RYGB (34). Different study groups have shown that BS improves and even reverses NAFLD in severely obese patients, on molecular, micro-, and macroscopic levels (6, 35–38). Unfortunately, we are not able to provide molecular analysis and follow-up analysis for the patients included. However, taking clinical parameters into consideration, we found that liver function remained stable in most patients over the follow-up period. Some patients experienced great improvement of the liver function and regression of NAFLD. Hence, they no longer remained on the waiting list for a LT.

In patients suffering from liver cirrhosis, BS may trigger acute-on-chronic liver failure warranting urgent LT (39–41). Iannelli et al. identified more patients with acute-on-chronic liver failure after BS, requiring high urgency LT (42). This emphasizes that BS, no matter which procedure is chosen, is a high-risk procedure in morbidly obese patients with liver cirrhosis (25). Most acute-on-chronic liver failures have been reported after RYGB and other more invasive procedures (39). Yet, Moulla et al. previously published a series of 25 patients, showing that BS, mainly RYGB, in high-risk patients is feasible without increased complication rates (21). However, when liver cirrhosis is discovered intraoperatively, prominent varices or technical difficulties may require a change of the operative procedure (31).

In order to reduce complications while improving liver function, the IFSO not only recommends preoperative TIPS, followed by an SG in NAFLD patients, but also evaluation of LT 3 months after SG. According to our experience, we agree with the IFSO on evaluating patients suffering from NAFLD for TIPS implantation and consecutive SG. However, in Germany, we are facing severe organ scarcity and would not be able to facilitate LT for many cases after BS in due time (43). These patients typically present with a low MELD score with a very long waiting time on the waiting list. In addition to organ scarcity, LT must be considered high-risk in this obese population (25). Taking these aspects into consideration, we would favor a reluctant approach toward LT and would rather wait for the effects of SG on weight and NAFLD cirrhosis and delist the patient from the LT waiting list whenever possible.

The current analysis has to deal with the limitations of a retrospective study and due to the sharp inclusion criteria with limitations of a very specific and small study population. Therefore, a multivariate statistical analysis is missing. Nonetheless, this study represents the experience of two high-volume bariatric centers with the possibility to perform LT. As obesity and NAFLD will become an imminent problem for healthcare providers in near future, further prospective analyses with a clear focus on perioperative risk evaluation, including identification of portal hypertension, are needed.

## Conclusion

Bariatric surgery can be performed safely in patients suffering from liver cirrhosis Child A. Furthermore, bariatric surgery may prevent progression of NAFLD and even reverse structural liver changes. However, patients suffering from NAFLD-associated liver fibrosis or cirrhosis need to be considered as severely sick and a careful risk profile analysis (Child–Pugh A, no portal hypertension) should be performed to proceed with BS. Preoperative TIPS implantation may reduce perioperative risk.

## Data Availability

Requests to access the datasets should be directed to the corresponding author.
